# Benefits of Intraaortic Balloon Support for Myocardial Infarction Patients in Severe Cardiogenic Shock Undergoing Coronary Revascularization

**DOI:** 10.1371/journal.pone.0160070

**Published:** 2016-08-02

**Authors:** Chun-Tai Mao, Jian-Liang Wang, Dong-Yi Chen, Ming-Lung Tsai, Yu-Sheng Lin, Wen-Jin Cherng, Chao-Hung Wang, Ming-Shien Wen, I-Chang Hsieh, Ming-Jui Hung, Chun-Chi Chen, Tien-Hsing Chen

**Affiliations:** 1 Division of Cardiology, Department of Internal Medicine, Chang Gung Memorial Hospital, Keelung, Taiwan, and Chang Gung University College of Medicine, Taoyuan, Taiwan; 2 Division of Cardiology, Landseed Hospital, Pingzhen City, Taiwan; 3 Division of Cardiology, Department of Internal Medicine, Chang Gung Memorial Hospital, Chang Gung University College of Medicine, Taoyuan, Taiwan; 4 Division of Cardiology, Department of Internal Medicine, Chang Gung Memorial Hospital, Chiayi, Taiwan, and Chang Gung University College of Medicine, Taoyuan, Taiwan; University of Tampere, FINLAND

## Abstract

**Background:**

Prior studies have suggested intraaortic balloon pump (IABP) have a neutral effect on acute myocardial infarction (AMI) patients with cardiogenic shock (CS). However, the effects of IABP on patients with severe CS remain unclear. We therefore investigated the benefits of IABP in AMI patients with severe CS undergoing coronary revascularization.

**Methods and Results:**

This study identified 14,088 adult patients with AMI and severe CS undergoing coronary revascularization from Taiwan’s National Health Insurance Research Database between January 1, 1997 and December 31, 2011, dividing them into the IABP group (n = 7044) and the Nonusers group (n = 7044) after propensity score matching to equalize confounding variables. The primary outcomes included myocardial infarction(MI), cerebrovascular accidents or cardiovascular death. In-hospital events including dialysis, stroke, pneumonia and sepsis were secondary outcomes. Primary outcomes were worse in the IABP group than in the Nonusers group in 1 month (Hazard ratio (HR) = 1.97, 95% confidence interval (CI) = 1.84–2.12). The MI rate was higher in the IABP group (HR = 1.44, 95% CI = 1.16–1.79), and the cardiovascular death was much higher in the IABP group (HR = 2.07, 95% CI = 1.92–2.23). The IABP users had lower incidence of dialysis (8.5% and 9.5%, P = 0.04), stroke (2.6% and 3.8%, P<0.001), pneumonia (13.9% and 16.5%, P<0.001) and sepsis (13.2% and 16%, P<0.001) during hospitalization than Nonusers.

**Conclusion:**

The use of IABP in patients with myocardial infarction and severe cardiogenic shock undergoing coronary revascularization did not improve the outcomes of recurrent myocardial infarction and cardiovascular death. However, it did reduce the incidence of dialysis, stroke, pneumonia and sepsis during hospitalization.

## Introduction

Acute myocardial infarction (AMI) complicated with cardiogenic shock (CS) is a complex syndrome which may induce low cardiac output and hypotension followed by multi-organ dysfunction [1]. Its mortality rate is between 40% to 60% even after early revascularization, including percutaneous coronary intervention (PCI) or coronary artery bypass grafting (CABG) [2–4]. In order to stabilize these critically ill patients, mechanical assistive devices such as intraaortic balloon pump (IABP), percutaneous left ventricular assist devices (for example, Impella) or venoarterial extracorporeal membrane oxygenationators (ECMO) have been developed to support hemodynamics, in addition to inotropic agents [5–7]. One mechanical assist device, IABP, has been used since 1968 [8], and has become a mature technology; it is the most common method of mechanical cardiac assistance used in acute cardiology today [9].

According to previous studies, IABP can increase diastolic coronary and systemic blood flow, and it reduces afterload and myocardial work, which is supposed to protect LV function and avoid low cardiac output [10,11]. A large national registry study with 23,810 patients in the US revealed that using IABP in patients with CS and AMI undergoing thrombolysis reduced in-hospital mortality by 18% [12], and a meta-analysis of cohort studies revealed that using IABP decreased 30-day mortality by 18% in these patients [9]. However, systemic review of randomized control trials (RCTs) revealed IABP did not improve outcomes either for patients undergoing thrombolysis or early revascularization [5,9]. Furthermore, the CRISP-AMI trial suggested that prophylactic use of IABP in AMI patients without CS does not improve cardiovascular outcomes either [13]. The benefits of IABP have thus become controversial. Then the overall use of IABP decreased significantly from 0.99% in 1998 to 0.36% in 2008 in the US [14]. In an effort to confirm its effect, a large well-designed RCT, IABP-SHOCK II, was performed. Its results showed that IABP may have less or no benefit for patients with AMI complicated by CS compared to standard therapy with inotropic agents in the following 30 days and one year [15,16].

According to the supplementary appendix of IABP-SHOCK II, over 95% of patients in the IABP group received inotropic agents to stabilize their hemodynamics [15]. The median dose of dopamine, norepinephrine and dobutamine administered to both groups was 4 ug/kg per minute, 0.3 ug/kg per minute and 10 ug/kg per minute [15]. The relatively lower doses of inotropic agents used and the relative lower mortality rate in one month (approximately 40%, as compared with 42% to 48% in other RCTs and registries) may indicate that this trial included a higher percentage of patients with mild or moderate CS, a factor that might preclude generalizing the results to patients with severe CS [2–4]. In order to evaluate the effect of IABP in patients with severe CS who need higher dose of inotropic agents, we design this study.

## Methods

### Study population

This study was a population-based cohort study using the 1997–2011 Taiwan National Health Insurance Research Database (NHIRD) released by the Taiwan National Health Research Institute (NHRI) (http://nhird.nhri.org.tw/en/index.htm), which consists of standard computerized claims documents submitted by medical institutions seeking reimbursement through the National Health Insurance (NHI) Program. During 2007, 98.4% of Taiwan’s approximately 22.96 million people were enrolled in this program. The accuracy and validation of NHIRD data are based on regular auditing of claims by the NHI Bureau [17,18]. False reimbursement claims result in substantial penalties. Minor infractions involve fines of 100 times the amount of the false claim, while serious infractions may result in revoking physicians’ licenses or criminal charges. This study was approved by the Ethics Institutional Review Board of Chang Gung Memorial Hospital (103-6077B).

### Analysis Cohort

We assembled a cohort of all adults in Taiwan who received a diagnosis of AMI between January 1, 1997 and December 31, 2011. The exclusion criteria were 1) patient age under 18 years old, 2) patients who did not receive revascularization such as PCI and CABG, 3) patients who did not fit the definition of severe CS, as described in the next section. Then they were divided into the IABP or Nonusers group according to the procedure codes used during the index admission. The final analysis cohort used for the primary end point consisted of all individuals meeting these criteria who had severe CS, as defined below.

### Definition of Severe Cardiogenic Shock

Severe CS was defined as receiving high dose dopamine during the index admission. According to the IABP-SHOCK II study, the median dosage of dopamine was around 4μg/kg per minute [15]. In the literature, over 5μg/kg per minute of dopamine is considered a high dose level [19]. Therefore, we defined severe CS as use of dopamine doses over 440mg (approximately 5μg/kg per minute for a 60kg adult for one day) to stabilize hemodynamics during the index admission. We have applied this inclusion criterion in our previous study [20].

### Study outcomes

The primary outcomes consisted of cardiovascular death, recurrent MI and cerebrovascular accident (CVA) during the follow-up period. Our definition of cardiovascular death meets the criteria of the Standardized Definitions for End Point Events in Cardiovascular Trials draft by the U.S. Food and Drug Administration [21]. Death and causes of death were determined according to the registry data in the NHIRD [17,18]. Repeat coronary revascularization during follow-up, including PCI and CABG, was also recorded. Each outcome of time to event was analyzed during a short-term follow up (1 month) and a long-term follow up (until the last follow up). Secondary outcomes (in-hospital events) included new onset of dialysis, stroke, pneumonia, intubation, sepsis and ECMO support during the index AMI hospitalization.

### Confounding variables

We took demographic factors including age and gender into account. Medical history, including prior MI, prior stroke, prior coronary intervention, prior carotid stenosis and prior peripheral artery disease, were identified by procedure codes and International Classification of Diseases, 9th Revision, Clinical Modification (ICD-9CM) diagnosis codes (S1 Table). Other comorbidities, the numbers of vessels targeted for intervention and dosage of dopamine and norepinephrine were also recorded (Table 1).

**Table 1:** Baseline characteristics of the patients.

	Before PSM matching			After PSM matching		
Characteristics	IABP	Nonuser	*P*	IABP	Nonuser	*P*
Patient number	9,295	12,711	—	7,044	7,044	—
Age (year)	69 [59, 77]	70 [59, 78]	0.003	70 [59, 77]	70 [59, 77]	0.947
Gender			<0.001			0.954
Male	6,980 (75.1)	9,069 (71.3)		5,201 (73.8)	5,204 (73.9)	
Female	2,315 (24.9)	3,642 (28.7)		1,843 (26.2)	1,840 (26.1)	
Prior myocardial infarction	1,728 (18.6)	2,594 (20.4)	0.001	1,366 (19.4)	1,390 (19.7)	0.610
Prior stroke	1,339 (14.4)	2,059 (16.2)	<0.001	1,044 (14.8)	1,082 (15.4)	0.371
Known peripheral arterial disease	645 (6.9)	1,041 (8.2)	0.001	504 (7.2)	523 (7.4)	0.538
Prior PCI	1,078 (11.6)	1,595 (12.5)	0.033	837 (11.9)	840 (11.9)	0.938
Prior CABG	114 (1.2)	158 (1.2)	0.913	80 (1.1)	83 (1.2)	0.813
Prior carotid stenting	13 (0.1)	9 (0.1)	0.109	7 (0.1)	7 (0.1)	1.000
Prior other Comorbidities						
Hypertension	4,480 (48.2)	7,111 (55.9)	<0.001	3,601 (51.1)	3,625 (51.5)	0.686
Dyslipidemia	1,827 (19.7)	3,430 (27.0)	<0.001	1,546 (21.9)	1,536 (21.8)	0.839
Diabetes mellitus	3,923 (42.2)	5,623 (44.2)	0.003	2,996 (42.5)	3,058 (43.4)	0.291
Coronary artery disease	2,214 (23.8)	3,313 (26.1)	<0.001	1,722 (24.4)	1,782 (25.3)	0.242
Heart failure	1,180 (12.7)	1,903 (15.0)	<0.001	958 (13.6)	975 (13.8)	0.677
Chronic kidney disease	622 (6.7)	1,171 (9.2)	<0.001	517 (7.3)	534 (7.6)	0.586
Dialysis	369 (4.0)	725 (5.7)	<0.001	315 (4.5)	341 (4.8)	0.299
Atrial fibrillation	678 (7.3)	1,008 (7.9)	0.080	532 (7.6)	558 (7.9)	0.412
Gout	604 (6.5)	988 (7.8)	<0.001	487 (6.9)	478 (6.8)	0.764
Chronic obstructive pulmonary disease	994 (10.7)	1,824 (14.3)	<0.001	855 (12.1)	848 (12.0)	0.856
Malignancy	457 (4.9)	755 (5.9)	0.001	377 (5.4)	352 (5.0)	0.342
No. of intervened disease vessels			<0.001			0.915
1	4,707 (50.6)	7,319 (57.6)		3,724 (52.9)	3,702 (52.6)	
2	1,952 (21.0)	2,213 (17.4)		1,373 (19.5)	1,374 (19.5)	
3	2,636 (28.4)	3,179 (25.0)		1,947 (27.6)	1,968 (27.9)	
Method of intervention			<0.001			0.397
PCI	5,873 (63.2)	8,846 (69.6)		4,534 (64.4)	4,582 (65.0)	
CABG	3,422 (36.8)	3,865 (30.4)		2,510 (35.6)	2,462 (35.0)	
Dosage of inotropic medication						
Dopamine (mg×10^3^)	2.4 [1.2, 5.2]	1.2 [0.8, 2.4]	<0.001	2.0 [0.8, 3.8]	2.0 [1.0, 3.6]	0.154
Norepinephrine (mg)	0 [0, 16]	0 [0, 4]	<0.001	0 [0, 8]	0 [0, 8]	0.212

### Propensity Score Matching Analysis

To simulate a randomized trial, we used propensity score matching (PSM) to reduce potential confounding and selection biases [18,22]. We estimated the propensity score for each patient by modeling the probability of receiving IABP given the following covariates: age, sex, history of MI, stroke, peripheral artery disease (PAD), PCI, CABG, and carotid stenting, other history of comorbidities such as hypertension, dyslipidemia, diabetes mellitus (DM), CAD, heart failure (HF), chronic kidney disease (CKD), dialysis, atrial fibrillation, gout, chronic obstructive pulmonary disease (COPD) and malignancy, numbers of intervened diseased vessels, and dose of dopamine and norepinephrine, as well as indexed year, which are all listed in Table 1. We subsequently used the derived propensity scores to match the 9,295 IABP users with 12,711 nonusers. The PSM matching algorithm was based on the nearest-neighbor method and used the caliper radius (set as 0.5 sigma), which signifies the tolerance level for the maximum distance in the propensity score. Finally, a total of 7,044 IABP users met the criteria. The matching procedure was performed with SAS Version 9.3 (SAS Institute, Cary, NC).

### Statistical Analysis

To compare clinical characteristics between study groups (IABP users and nonusers), we used chi-square test for categorical variables and independent sample t-test for continuous variables. We compared time to the first event of composite primary outcomes (as well as revascularization outcomes) after index hospitalization between study groups using multivariable Cox proportional hazard models with adjustment of the propensity score. The incidence of secondary outcomes (in-hospital events during the index hospitalization) between study groups was compared by multivariable logistic regression analysis with adjustment of the propensity score. All data analysis used IBM SPSS software version 22 (IBM SPSS Inc, Chicago, Illinois).

## Results

### Patient characteristics

**Fig 1:**
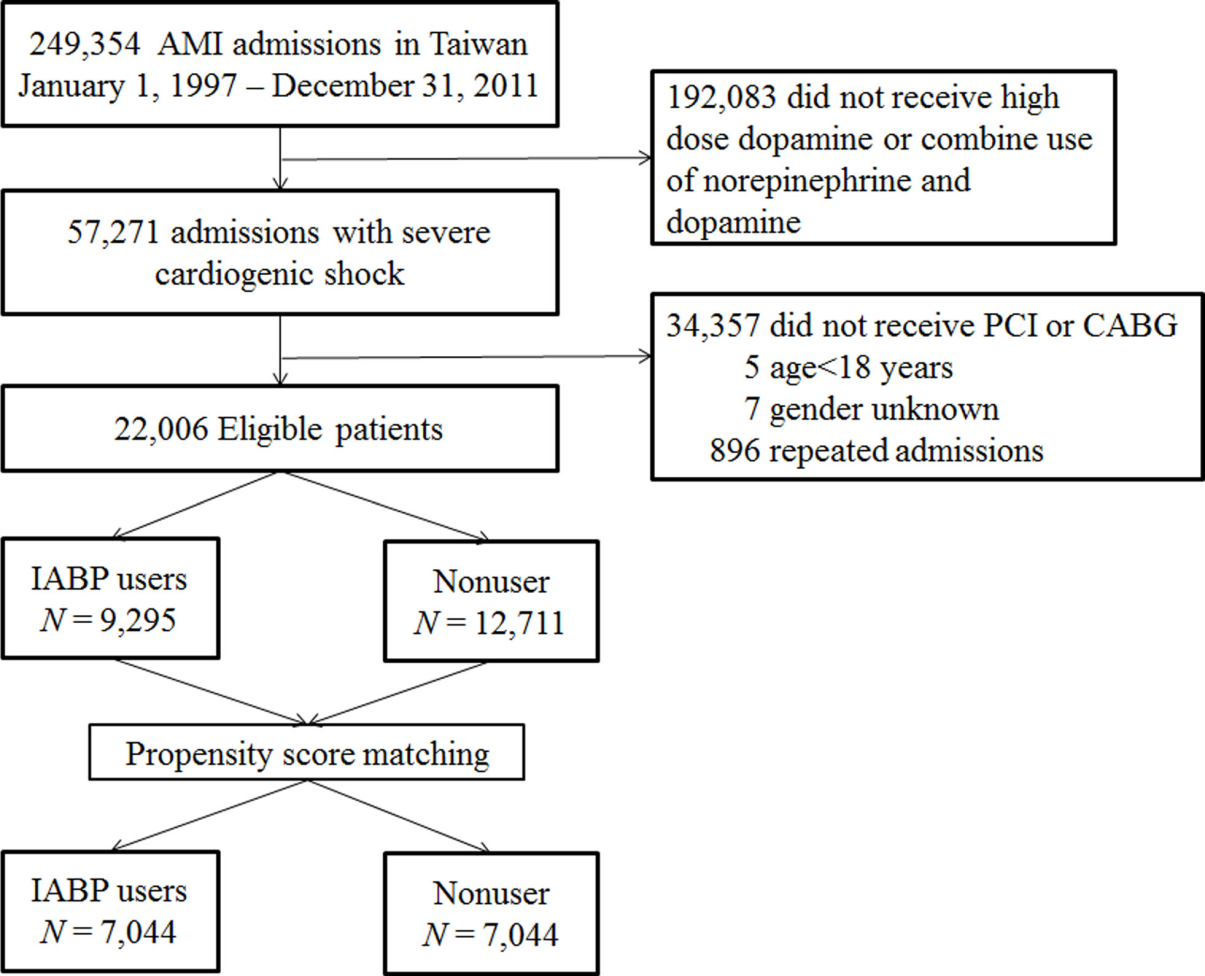
Flowchart of Inclusion. Individuals with AMI and severe cardiogenic shock undergoing coronary revascularization were included in our analysis after relevant exclusions (AMI = acute myocardial infarction, PCI = percutaneous coronary intervention, CABG = coronary artery bypass surgery, IABP = intraaortic balloon pump).

Table 1 demonstrates the baseline patient characteristics before and after PSM analysis. The study identified a total of 249,354 patients who were admitted for AMI. We excluded 192,083 patients who did not meet the definition of severe CS, 34,357 patients who did not receive revascularization, 896 patients with repeated admissions, 5 patients of young age (<18 years) and 7 patients whose gender was unknown. The final analysis cohort consisted of 22,006 adult patients, of whom 9,295 were treated with IABP and 12,711 were treated without IABP (Fig 1).

Before PSM analysis, patients who received IABP treatment were more likely than nonusers to be younger and male. These patients were less likely to have history of MI, stroke and PAD, and less likely to have received PCI before. In addition, they were also less likely to have hypertension, dyslipidemia, DM, CAD, HF, CKD, dialysis, gout, COPD and malignancies. However, these patients were prescribed higher doses of dopamine. After PSM matching, 7,044 IABP users were successfully matched to nonusers (Fig 1). The distribution of clinical characteristics between IABP users and nonusers was similar after PSM matching. The median age in both groups was 70 years. About 73% of patients were male. 64.4% of patients in IABP group received PCI for the treatment of AMI with cardiogenic shock and 65% of patients in Nonuser group received PCI. There was no difference between two groups. The median dose of dopamine given to IABP users and nonusers of IABP was 2000 mg, which was equal to 7.7μg/kg per minute for a 60 kg patient over three days. The median follow up time was 0.7 years in IABP users and 1.6 years in nonusers.

### Primary outcomes during the short-term and long-term follow-up

Table 2 displays the results of primary outcomes. Composite primary outcome events occurred in 3187 patients (45.2%) in the IABP group and 2403 patients (34.1%) in the Nonuser group (Hazard ratio (HR) = 1.56, 95% confidence interval (CI) = 1.48–1.64, p<0.001). We found a higher cardiovascular death rate in the IABP group than the Nonuser group (36.9% versus 24%, p<0.001), and a higher recurrent MI rate in the IABP group than the Nonuser group (7% versus 6.9%, p = 0.001); however, the rate of CVA was similar between the two groups (HR = 1.03, 95% CI = 0.9–1.19, p = 0.654).

**Table 2:** Primary outcomes in the short-term and long-term follow-up periods.

	Number of event (%)		HR (95% CI)*
Outcome	IABP	Nonuser	
	(*n* = 7,044)	(*n* = 7,044)	
**1 month follow up**			
Myocardial infarction	192 (2.7)	143 (2.0)	1.44 (1.16–1.79)
Cerebrovascular accident	39 (0.6)	35 (0.5)	1.23 (0.78–1.94)
Cardiovascular death	1,953 (27.7)	1,050 (14.9)	2.07 (1.92–2.23)
Primary composite endpoint§	2,143 (30.4)	1,215 (17.2)	1.97 (1.84–2.12)
**All course (the last follow up)**			
Myocardial infarction	496 (7.0)	486 (6.9)	1.24 (1.10–1.41)
Cerebrovascular accident	355 (5.0)	443 (6.3)	1.03 (0.90–1.19)
Cardiovascular death	2,600 (36.9)	1,690 (24.0)	1.76 (1.66–1.87)
Primary composite endpoint	3,187 (45.2)	2,403 (34.1)	1.56 (1.48–1.64)

HR = hazard ratio; CI = confidence interval

§ The follow-up rate at 1 month was 63.9% (4,498) and 79.0% (5,562) in the IABP and Nonuser, respectively.

* Adjusted by propensity score.

Secondary outcomes (in-hospital event during the index AMI hospitalization)

The results for in-hospital events are revealed in Table 3. Patients in the IABP group had lower risk of new onset of dialysis and stroke (OR = 0.88, 95% CI = 0.79–0.99; 0R = 0.65, 95%CI = 0.54–0.79, respectively), compared to those in the Nonuser group. In addition, patients treated with IABP had a lower chance of getting pneumonia and sepsis than those who didn’t receive IABP (OR = 0.82, 95% CI = 0.74–0.90; OR = 0.79, 95%CI = 0.72–0.87, respectively). IABP use also did not increase the amputation rate (OR = 1.18, 95% CI = 0.79–1.77).

**Table 3:** In-hospital events during the index AMI hospitalization.

Outcome	Number of event (%)		OR (95% CI)*
	IABP	Nonuser	
	(*n* = 7,044)	(*n* = 7,044)	
New onset of dialysis	599 (8.5)	667 (9.5)	0.88 (0.79–0.99)
New onset of stroke	180 (2.6)	271 (3.8)	0.65 (0.54–0.79)
New onset of hemorrhagic stroke	33 (0.5)	43 (0.6)	0.77 (0.49–1.21)
New onset of ischemic stroke	158 (2.2)	262 (3.7)	0.59 (0.49–0.72)
Pneumonia	981 (13.9)	1,161 (16.5)	0.82 (0.74–0.90)
Intubation	3,173 (45.0)	2,537 (36.0)	1.49 (1.39–1.59)
Sepsis	928 (13.2)	1,126 (16.0)	0.79 (0.72–0.87)
Amputation	52 (0.7)	44 (0.6)	1.18 (0.79–1.77)
ECMO support	459 (6.5)	123 (1.7)	4.02 (3.28–4.92)

OR = odds ratio; CI = confidence interval

* Adjusted by propensity score.

The results of revascularization are shown in Table 4. The occurrence of repeat PCI in 30 days was similar in the IABP group and the Nonuser group (HR = 1.17, 95% CI = 0.92–1.48); however, more patients needed further CABG in the IABP group than in the Nonuser group (HR = 2.41, 95% CI = 1.62–3.57). During the follow-up period, patients treated with IABP were at greater risk of receiving repeated revascularization than those who did not receive IABP treatment (HR = 1.19, 95% CI = 1.10–1.28).

**Table 4:** Revascularization in the short-term and long-term follow-up periods.

Outcome	Number of event (%)		HR (95% CI)*
	IABP	Nonuser	
	(*n* = 7,044)	(*n* = 7,044)	
**1 month follow up**			
PCI	137 (1.9)	137 (1.9)	1.17 (0.92–1.48)
CABG	80 (1.1)	36 (0.5)	2.41 (1.62–3.57)
Total revascularization**§**	210 (3.0)	168 (2.4)	1.43 (1.17–1.76)
**All course (the last follow up)**			
PCI	1,149 (16.3)	1,294 (18.4)	1.14 (1.05–1.23)
CABG	209 (3.0)	167 (2.4)	1.54 (1.25–1.88)
Total revascularization	1,283 (18.2)	1,390 (19.7)	1.19 (1.10–1.28)

PCI: percutaneous coronary intervention; CABG: coronary artery bypass grafting surgery

HR = hazard ratio; CI = confidence interval

§ The follow-up rate at 1 month was 63.9% (4,502) and 79.0% (5,566) in the IABP and Nonuser, respectively.

* Adjusted by propensity score.

## Discussion

This study is based on the analysis of a nationwide database in Taiwan. Our results demonstrate that IABP did not improve cardiovascular outcomes, including recurrent MI, CVA or cardiovascular death, in patients with severe CS undergoing coronary revascularization, not at 30 days or over the full course of follow up. Furthermore, we found a higher incidence of recurrent MI and cardiovascular death in IABP users. On the other hand, IABP unexpectedly decreased in-hospital events including dialysis, new onset of stroke, pneumonia and sepsis, a finding not identified by previous studies [15,23]. Using IABP remains a reasonable treatment choice for patients with profoundly unstable hemodynamics as it reduces in-hospital events, though not mortality. Combination therapy using other mechanical assistive devices with IABP may provide better outcomes.

**Fig 2:**
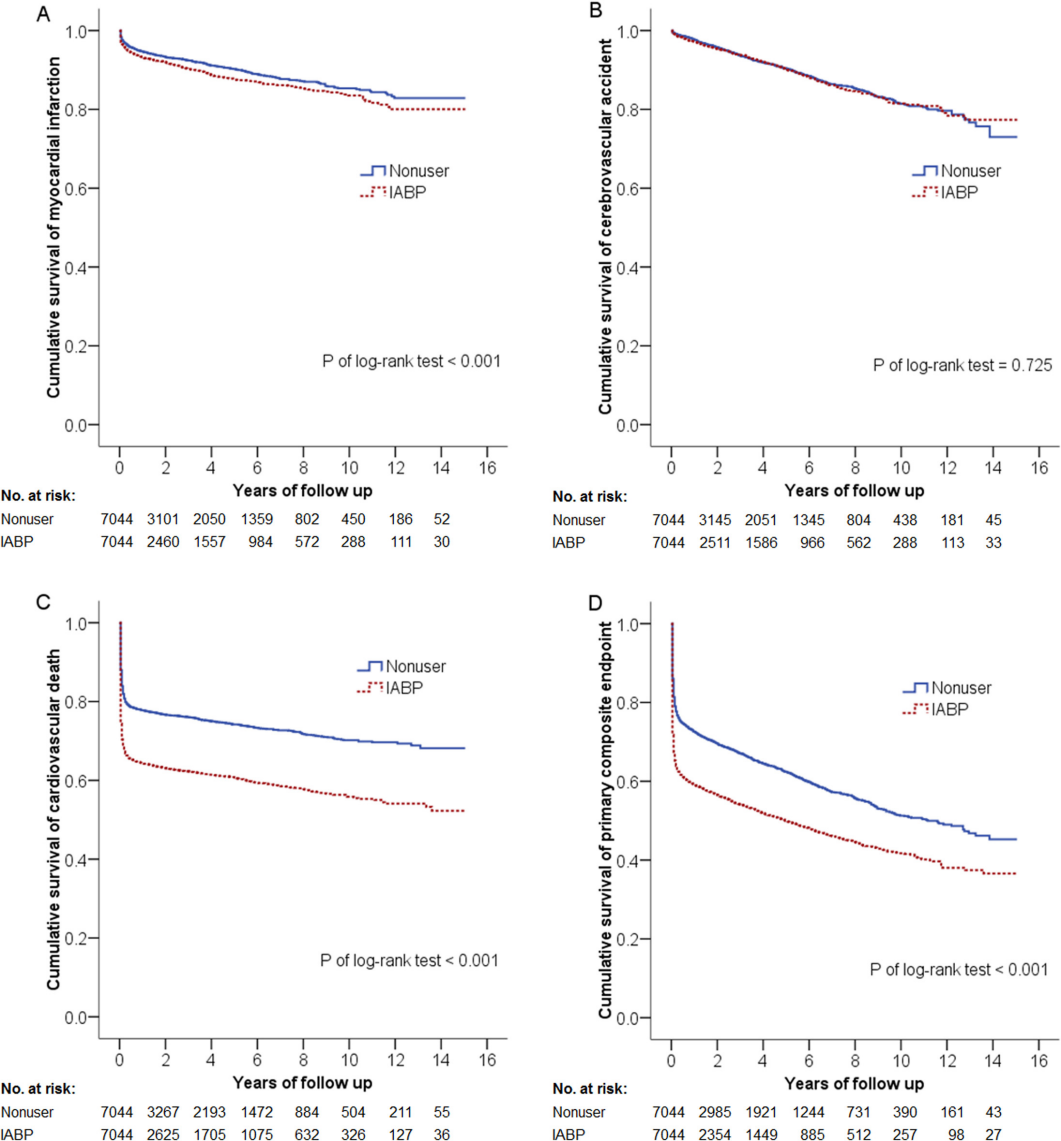
**Cumulative probability of event-free survival for (A) myocardial infarction, (B) cerebrovascular accident, (C) cardiovascular death, and (D) primary composite endpoint.** The primary outcome was a composite of myocardial infarction (MI), cerebrovascular accident (CVA), or cardiovascular death. A higher recurrent MI rate in the IABP group than the Nonuser group (7% versus 6.9%, p = 0.001). The rate of CVA was similar between the two groups (5% versus 6.3%, p = 0.654). A higher cardiovascular death rate in the IABP group than the Nonuser group (36.9% versus 24%, p<0.001). More composite primary outcome occurred in IABP group than Nonuser group (45.2% versus 34.1%, p<0.001).

In our study, patients received approximately twice as much dopamine as patients in the IABP-SHOCK II study (7.7 mcg/kg per minute versus 4 mcg/kg per minute). Under these worse circumstances, the more unstable hemodynamics is reasonably considered in patients with IABP treatment than those without IABP treatment. Besides higher rates of repeat revascularization and higher incidence of CABG in the IABP group may indicate that IABP users might have worse coronary artery condition than nonusers. More unstable hemodynamic status and critical coronary lesions may cause poorer primary composite outcomes in IABP users, especially cardiovascular outcomes, including recurrent MI and cardiovascular death (Fig 2). Our findings are also consistent with those of a US study from the Nationwide Inpatient Sample Database which suggested that patients who received IABP had substantially higher risk of shock compared with those who did not receive IABP, and it was associated with markedly higher mortality rates [14]. Although IABP can improve coronary flow and reduce afterload [10,11,24], it could not mitigate the poor outcomes in patients with severe CS. Then other mechanical assist devices such as ECMO and percutaneous LVAD were developed to provide stronger hemodynamic support [7,25]. Nevertheless, a meta-analysis of controlled trials showed that percutaneous LVAD did not improve 30-day mortality compared to IABP and there are no randomized study data available for use of ECMO in patients with AMI and CS [26,27]. Therefore, it is reasonable that combination therapy incorporating IABP and other mechanical assistive devices might be a useful solution. Unlike venoarterial ECMO which provides continuous and backward blood flow from the descending aorta to the left ventricle, IABP can augment forward pulsatile blood flow according to the cardiac cycle. The combined use of these two devices may offer greater benefits to patients with severe CS [28–30]. Moreover, a substudy of the CRISP-AMI trial also revealed that IABP decreased six-month mortality in large anterior myocardial infarctions complicated by persistent ischemia after PCI [31]. IABP may still have a role in the treatment of compromised hemodynamics and complicated CAD.

According to the IABP-SHOCK and IABP-SHOCK II trial, using IABP in patients with CS did not improve renal function or other in-hospital events [15,23], but in our study, patients receiving IABP had lower incidence of new dialysis than those who did not receive IABP. Previous studies demonstrated that IABP can increase eGFR by approximately 16 mL/min/1.73m2 [32,33]. LVAD combined with IABP may preserve more renal function [34]. This renal protection effect may be more important for patients with severe CS than those with mild to moderate CS to decrease the likelihood of needing dialysis. Moreover, IABP can also enhance approximately 16 to 43% of cerebral blood flow in human beings [35,36]. This neuroprotective effect may be the reason for the decreased incidence of stroke during hospitalization among the IABP users in our study. However, there was no significant difference between the two groups in overall risk of CVA in long-term follow up. On the other hand, our study suggested lower incidence of pneumonia and sepsis in IABP users. In the SHOCK trial, median systemic vascular resistance (SVR) was within the normal range under vasopressor therapy during CS, which means that SVR may be low in some patients, similar to septic shock [37]. In fact, sepsis was suspected in 18% of the SHOCK trial cohort, 74% of whom developed positive bacterial cultures [37]. These findings are consistent with the observation that MI can cause systemic inflammatory response syndrome (SIRS) and suggest that inappropriate vasodilation, which is an aspect of SIRS, results in impaired perfusion of the intestinal tract, enabling transmigration of bacteria and sepsis. Interlukin-6, tumor necrosis factor-α and other circulating factors (complement, procalcitonin, neopterin, C-reactive protein, and others) have been reported to contribute to SIRS in CS [1,38,39]. IABP improves coronary and peripheral perfusion via diastolic balloon inflation and augments LV performance via systolic balloon deflation with an acute decrease in afterload, which may mitigate the severity of SIRS and consequently decrease the risk of sepsis [1].

Our results suggest that IABP has indisputable hemodynamic benefits in patients with severe CS, which include reducing the incidence of dialysis, stroke, pneumonia and sepsis. It may still be reasonable to use IABP in patients with severe CS even if it does not ultimately lower mortality. Therefore, using IABP in the treatment of cardiogenic shock is the class IIa recommendation in the American Heart Association and American College of Cardiology practice guidelines; nevertheless, it is the class IIb recommendation in ESC guideline [40,41]. In the meanwhile, further investigation of the role of IABP and combination therapy with other mechanical assistive devices in the treatment of complicated coronary artery disease and CS is merited.

## Study Limitations

Our study, based on data retrieved from Taiwan’s NHIRD, may provide useful information about the effects of IABP in AMI patients with severe CS, but it nonetheless has some limitations. First, Taiwan’s NHIRD does not record certain types of personal information for our patients, such as family history of cardiovascular disease, lifestyle or laboratory parameters, including serum lactate and B-type natriuretic peptide (BNP), which could contribute to a more detailed analysis. Furthermore, the scoring system for severity of coronary artery disease such as SYNTAX score was unavailable in NHIRD and this database only notes the events of revascularization without etiology or location of culprit lesions, so it was difficult to distinguish between target lesion revascularization/target vessel revascularization and staged revascularization. In the meantime, we may include few amount of very ill patients suffering from AMI, pneumonia or other sepsis complicated with cardiogenic and septic shock undergoing treatment of high dose inotropic agents. Besides we cannot clarify the treatment duration of dopamine, the concentration of dopamine solution and the adjusted dosage of dopamine during the treatment course, whereas it is reasonable to use a total dose of dopamine for evaluation of disease severity. Therefore, we have applied this model in our previous study [20]. Second, the quality of data in the NHIRD is dependent upon the accuracy and completeness of documentation and abstraction. Potential confounding introduced by overcoding or undercoding cannot be completely eliminated; however, Taiwan’s NHI uses a systematic formula for auditing claims and the penalties for false claims are high. The data in the NHIRD are considered to be reliable and validated and have been used in many prior studies [17,18]. Third, we cannot exclude the possibility that residual measured and unmeasured confounding variables might account for differences we observed despite well PSM analysis and multivariable adjustment. Fourth, selection bias affecting physician decision-making and IABP placement decisions may affect our findings although we did adjust for multiple baseline differences.

## Conclusion

The use of intraaortic balloon pump in patients with myocardial infarction and severe cardiogenic shock undergoing coronary revascularization did not improve the outcomes of recurrent myocardial infarction, cerebrovascular accident, and cardiovascular death during the follow-up periods. However, it did reduce the incidence of dialysis, stroke, pneumonia and sepsis during hospitalization.

## Supporting Information

S1 Table ICD-9-CM code used for diagnosis or treatment in the current study. (DOCX)
