# Supplementary material for: Benefits of Intraaortic Balloon Support for Myocardial Infarction Patients in Severe Cardiogenic Shock Undergoing Coronary Revascularization
Source: PLoS One. 2016 Aug 2;11(8):e0160070. doi: 10.1371/journal.pone.0160070 (PMC4970797; doi:10.1371/journal.pone.0160070)
Supplement: S1 Table — (DOCX) [file pone.0160070.s001.docx]

S1 table. ICD-9-CM code used for diagnosis or treatment in the current study

| Variable | Source | Code |
| --- | --- | --- |
| Acute myocardial infarction | ICD-9 CM | 410.xx, 412.xx |
| Stroke | ICD-9 CM | 430.xx–436.xx |
| Peripheral arterial disease | ICD-9 CM | 440.0, 440.2x, 440.3x, 440.4, 440.9, 443.9, 444.2, 444.22, 444.8, 444.81,  445.0, 445.02, 250.7x, 707.1x |
| Hypertension | ICD-9 CM | 401.xx–405.xx |
| Dyslipidemia | ICD-9 CM | 272.xx |
| Diabetes mellitus | ICD-9 CM | 250.xx |
| Coronary artery disease | ICD-9 CM | 413.xx,414.0x |
| Heart failure | ICD-9 CM | 428.xx |
| Chronic kidney disease | ICD-9 CM | 585 |
| Dialysis | 1. ICD-9 CM in HV file;  2. Order code | 1. 585  2. 58001C,58027C |
| Atrial fibrillation | ICD-9 CM | 42731 |
| Gout | ICD-9 CM | 274.xx |
| Chronic obstructive pulmonary disease | ICD-9 CM | 490.xx–496.xx |
| Malignancy | ICD-9 CM | 140.xx–208.xx |
| Acute renal failure | ICD-9 CM | 584.xx |
| Pneumonia | ICD-9 CM | 480.xx–486.xx, 5070 |
| Sepsis | ICD-9 CM | 038.xx, 5990, 7854, 99662, 99762, 7070, 44024, 6826, 73027, 72886, 7907, 59010 |
